# Communicating through scents: an interview with Jane Hurst

**DOI:** 10.1186/s12915-018-0596-2

**Published:** 2018-11-01

**Authors:** Jane Hurst

**Affiliations:** Mammalian Behaviour & Evolution Group, Institute of Integrative Biology, University of Liverpool, Liverpool, UK

**Keywords:** Behaviours, Scents, Olfactory communication, MUPs, Darcin

## Abstract

Jane Hurst is a William Prescott Professor of Animal Science at the University of Liverpool, UK, studying scent communication in mammals and its role in behaviours. In this interview, Jane discusses how scents encode complex information in rodents, driving behaviours such as kinship interactions and choosing a mate, how understanding natural behaviours of animals can inform experimental designs, and what is the connection between Jane Austin and pheromones.

## What are the questions driving your research?

I am interested in the social organization of animals and in the communication that mediates their interactions. Much of my research has focused on scent communication among mammals, which can be extremely complex, with hundreds of component molecules.

The complexity of this system and behaviours raises many questions. For instance, what is the information communicated through these complex scents? Animals can discriminate between individuals based on scent, and recognize similarity in the scents of closely related individuals. Is this based simply on the overall similarity of body odours correlating with genetic similarity, or have animals evolved specific scent components that signal individual identity and kinship? Does this differ between social and non-social species? Do mammals use simple pheromone signals to stimulate specific responses in conspecifics—like invertebrate pheromones—or do they use the information gained through scents in a more sophisticated way that allows them to modify their responses according to individual experience and learning? Individuals often differ in the investment that they make in particular components of scents—what information does this signal?

Studies so far have focused largely on males—are there major differences between males and females in scent use, and how does this differ between social systems? Also, most studies have focused on the volatile components of scents, but we now understand that involatile proteins and peptides play a wide variety of roles as well. What are the selection processes determining the evolution of scent components, and how do these different components interact? Finally, can we exploit the scent signals that mammals use to improve their management, particularly for more effective pest control?

**Fig. 1.**
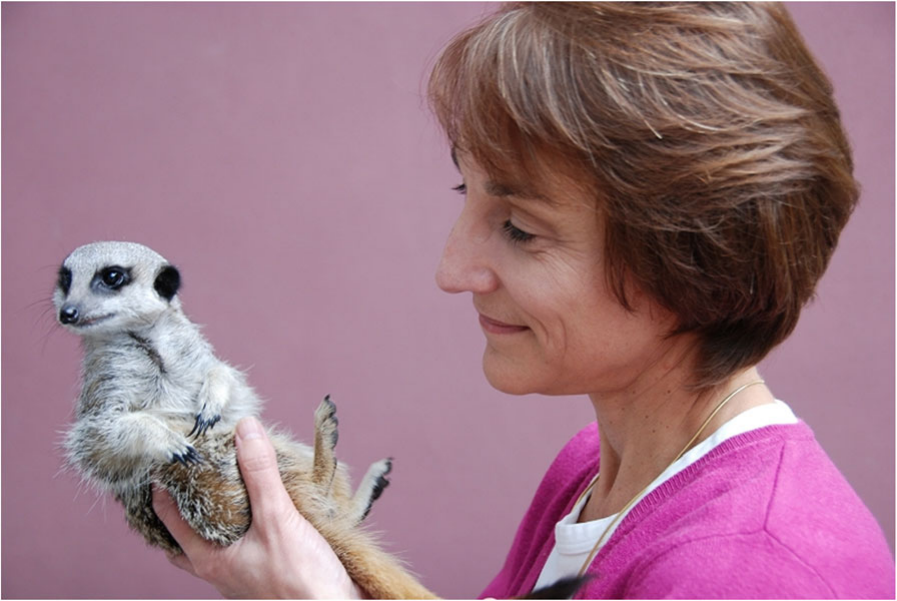
ᅟ

## You published several papers in *BMC Biology*, including one that brought us a protein named darcin, after a Jane Austin hero. Can you tell us a little bit about these studies?

We wanted to understand signals in scent that underpin mate choice: how animals assess potential mates, and how they communicate that information. We use wild house mice as an experimental model to ensure normal genetic variation, as genetic compatibility, heterozygosity and the ability to discriminate between individuals are all important components of mate choice. Wild house mice also appear to be a lot choosier when selecting a mate than domesticated laboratory mice, which is perhaps not surprising as laboratory strains have been artificially selected to breed easily.

Several androgen-dependent volatile pheromones had been identified in male mouse urine and it was generally assumed that females were attracted by these odours. However, we were surprised to find that females were no more attracted to male airborne odour than to equivalent odour from another female, unless they had direct contact with a male’s urine. This suggested that something involatile present in the male urine is essential for the sexual attraction. But we also discovered that just a few seconds of contact with a male’s urine stimulated females to learn a strong attraction to airborne odour from that particular male. That led us to the hypothesis that there is an involatile pheromone in male urine that females find highly attractive. And, through a process of rapid associative learning (similar to learning by Pavlov’s dogs but much quicker), this pheromone also stimulates subsequent attraction to the airborne odour signature of that individual male [1]. Through subsequent analyses we found that this involatile signal belonged to a family of major urinary proteins (MUPs).

Because of its key importance in determining female attraction to a male’s odour, we named the pheromone darcin (a more memorable name than its official MGI nomenclature as MUP20). While Jane Austen perceptively recognised that “*it is a truth universally acknowledged that a single man in possession of a good fortune must be in want of a wife*” [2], as far as male mice are concerned, it seems that a single mouse in possession of darcin must be in want of a mate.

Our darcin studies revealed a new mechanism of pheromone-induced learning that modifies responses to different individuals. Later on, we found that contact with darcin stimulates not only short- but also long term-learning [3] and leads to changes in the brain, increasing neurogenesis in both a female’s hippocampus and olfactory bulbs [4]. Most recently, we’ve shown that darcin, combined with other MUPs in mouse urine, shapes the individual odour signature that females learn when they contact a male’s scent [5].

## Looking back, is there a project that your lab pursued that stands out for you as particularly inspiring, tough or simply memorable?

Our darcin studies have been particularly inspiring from a personal perspective because they have changed my thinking about the contribution of pheromones to learned behaviour and show how pheromones and signatures of identity in scents work in concert.

But one of our most memorable studies was when we first tried to use laboratory mice—in this case inbred MHC-congenic strains—as a model for wild mice, in order to identify the genetic basis of scents that underlie the recognition of individual competitors. After years of working with genetically heterogeneous wild rodents, which can be difficult to handle unless approached very carefully, I thought that working with domesticated laboratory strains would be a piece of cake. How wrong I was! The caged laboratory mice would not reliably show the normal competitive scent marking behaviour typical of our captive wild house mice, so we decided to set them up in floor enclosures like our wild mice to promote more natural behaviour. As they seemed very timid, we put their home cages in the enclosures and opened the cage lids, leaving them to explore the enclosures at their own pace. Wild mice normally take a few minutes to settle down and start to explore, but the laboratory mice were reluctant to leave their cages and we had to put in ladders to encourage them to climb down and explore! After a few weeks in the enclosures, we eventually got the competitive scent marking that we were looking for but, even then, needed sample sizes that were much greater than we ever needed for wild mice, despite their genetic homogeneity. The unexpected discovery from these studies was that laboratory mice can be far more anxious than I expected to see from domesticated animals. I wanted to understand why they were so anxious and, much to my surprise, I discovered that it is because of the standard way that laboratory mice are handled. Almost universally, they are picked up by the tail, but it turns out that this is strongly aversive to mice and stimulates high anxiety. In a series of studies, we have shown that using non-aversive handling methods to pick up mice stimulates much less anxiety [6, 7] and that mice handled that way can be significantly more reliable in behavioural testing [8]. Picking up mice using a handling tunnel—a method we developed to handle wild rodents with minimal stress so that they will show naturalistic behaviour in captivity—works particularly well. Scooping up laboratory mice on the open hand is also less aversive. Studies in other laboratories have confirmed our findings and have also shown that using our non-aversive handling methods improves reward-based learning [9] and physiological responses such as glucose tolerance [10]. Because picking mice up by the tail has such a strong negative impact on mice and is likely to be a significant confounding factor in studies, we have made a freely available tutorial and other resources for implementing non-aversive handling methods for normal mouse husbandry and during experiments (see https://www.nc3rs.org.uk/how-to-pick-up-a-mouse).

## Is there a paper or a scientist that inspired you, or was seminal for your research?

The inspiration for my research comes first and foremost from watching animals, a fascination I developed from a very early age. But the inspiration to study the behavioural ecology of wild house mice for my PhD came from reading a book called ‘Mice All Over’ by Peter Crowcroft (published in 1966 by Foulis, London). After the Second World War, large grain stores were used to stockpile food reserves. These were a magnet to house mice, which were causing substantial spoilage. So Peter Crowcroft and Fred Rowe, working on behalf of the UK Government, set about trying to understand their behaviour to be able to control these problems more effectively. Crowcroft wrote not only about their findings but also about how they went about their studies, and I was hooked by the idea that my fascination with animal behaviour could contribute to research and also have a useful purpose. I was particularly intrigued by his accounts of a species so flexible and successful in exploiting human resources, outwitting our attempts to control them. So I chose to spend many hours during my PhD watching wild house mice in agricultural buildings such as poultry houses, trying to understand their social organization and how they were able to sustain much higher population densities than other mammal species. While poultry houses did not strike me as a great environment from my own personal perspective, these were clearly great for mice.

It was during these studies that I became interested in scent communication. While watching the animals through infrared cameras, it became clear that they were gaining a considerable amount of information through their noses, both from the numerous scent marks that they were depositing around their territories and when they interacted with each other. I realized that I would have to understand these scent signals to understand their behaviour. However, scent communication was a very under-researched area despite scents providing perhaps the most widespread means of communication between animals. This lack of knowledge, particularly in mammals, inspired me to start my research in this area.

**Website:** https://www.liverpool.ac.uk/mbe/members/Hurst/Hurst.html
